# One-Year Results of a Synthetic Intervention Model for the Primary Prevention of T2D among Elderly Individuals with Prediabetes in Rural China

**DOI:** 10.3390/ijerph14040417

**Published:** 2017-04-14

**Authors:** Zhao Hu, Lulu Qin, Huilan Xu

**Affiliations:** 1 Department of Social Medicine and Health Management, Xiangya School of Public Health, Central South University, Changsha 410078, China; 15200807487@163.com (Z.H.); powerestlulu@163.com (L.Q.); 2 Department of Preventive Medicine, Medical School, Hunan University of Chinese Medicine, Changsha 410208, China

**Keywords:** pre-diabetes, intervention, physical activity

## Abstract

*Objective*: The objective of this study was to evaluate the effectiveness of a synthetic intervention model aimed at preventing type 2 diabetes and controlling plasma glucose, body weight and waist circumference in elderly individuals with prediabetes in rural China. *Methods:* We randomly assigned 434 (180 men and 254 women; mean age, 69 years; mean body mass index, 23.6 kg/m^2^) with prediabetes to either the intervention group or the control group. Each participant in the intervention group received synthetic intervention for 1 year. *Results:* The incidence of diabetes was 4.2% in the intervention group, versus 19.7% in the control group at the end of 1 year (*p* < 0.001). Compared with the control group, the intervention group experienced a great decrease in fasting glucose (−3.9 vs. 2.2 mg/dL, *p* < 0.001), body weight (−3.2 vs. 1.7 kg, *p* < 0.001), waist circumference (−2.4 vs. 1.0 cm, *p* < 0001), total cholesterol (−9.1 vs. −4.6 mg/dL. *p* = 0.014) and HbA1c (−1.0 vs. 0.1 mg %, *p* = 0.002) at the end of 1 year. *Conclusions*: The incidence of diabetes of the control group was higher than that of the intervention group. Besides, the synthetic intervention contributes to weight loss and glucose decrease, and may be effective in reducing the risk of diabetes among elderly individuals with prediabetes in rural China.

## 1. Introduction

Over the past several decades, the prevalence of type 2 diabetes has been very high and has increased worldwide [1]. Many follow-up studies have identified that people with prediabetes were at a high risk of developing diabetes [2,3].

Prediabetes is an intermediate category between normal glucose tolerance and overt diabetes and has been classified into three types: impaired fasting glucose (IFG), impaired glucose tolerance (IGT), and IFG combined with IGT. Approximately 5%–10% of people with prediabetes become diabetic annually, although the conversion rate varies by population and the definition of prediabetes [4,5]. Therefore, people with prediabetes are an important target group for interventions intended to prevent diabetes.

Several trials have demonstrated that there was a reduction in the risk of developing diabetes among prediabetic individuals after lifestyle and drug-based interventions [6,7,8,9]. The Diabetes Prevention Program (DPP) [8] showed that lifestyle intervention reduced the incidence by 58% (95% CI: 48% to 66%) after 2.8 years of continuous intervention for prediabetes. The Finnish Diabetes Prevention Study (DPS) [9] found that the cumulative incidence of diabetes was 11% (95% CI: 6% to 15%) in the intervention group and 23% (95% CI: 17% to 29%) in the control group after four years of lifestyle intervention for IGT. Similar results were obtained in other studies, such as the Malmo study [10] in Sweden and the Indian Diabetes Prevention Program [11]. In addition to reducing the incidence of diabetes, they also reduced the risk of adiposity. Weight loss was the dominant predictor of a reduced diabetes incidence. Some studies demonstrated that target weight reduction interventions could reduce the diabetes risk. For example, Hamman et al. showed that there was a 16% reduction in risk for every kilogram of weight loss after adjusting for changes in diet and activity [12], and a study published in 2013 showed that IGT patients with ≥5% weight loss at one year had a 65% lower incidence of T2D (HR 0.35, 95% CI: 0.22 to 0.56) [13].

However, most of these studies were conducted in resource-intensive settings [14] with selected volunteers, and translation studies have been implemented in real-life settings to evaluate their effectiveness. These studies focused on the long-term effects of the approaches on the prevention of diabetes and metabolic syndrome, but these interventions may not work in some other areas, especially in rural areas, due to differences in the social, economic and cultural forces [15]. 

In China, it is estimated that more than 92.4 million adults have diabetes and 148.2 million adults have prediabetes [16]. The occurrence of diabetes and prediabetes increases with increasing age. Almost 20.4% and 25.0% of the elderly (age 60 years and above) have diabetes and prediabetes, respectively, in China [16]. It has been demonstrated that prediabetes is likely to progress to diabetes within 10 years without timely and effective intervention [17].

To date, the effectiveness of interventions for the prevention of diabetes has not been investigated among the elderly in rural China. In the Da Qing IGT and Diabetes Study [18,19], the age of the study subjects was 45 years old on average, and the study was conducted in an industrial urban area. The results of that 20-year study may not be applicable to a rural population because urban residents have a higher prevalence of prediabetes owing to their different economic and social conditions. Moreover, the authors have stated that further studies are needed in other ethnic and socioeconomic groups to identify the most appropriate intervention strategies. We, therefore, conducted this study in rural prediabetic elderly residents to explore whether a synthetic intervention model can reduce the incidence of diabetes and to improve the metabolic outcome. 

## 2. Materials and Methods 

### 2.1. Research Design 

A randomized controlled trial had been conducted in the rural areas of Yiyang City of Hunan Province (China) from April 2015 to July 2016. Subjects who were diagnosed as prediabetic were aged 60 years and above. This was a community-based prospective study and the study groups were classified as followed: (1) Control group: prediabetics who were given standard health advice; (2) Intervention Group: prediabetics who were given an intense synthetic intervention. This study was approved by the IRB of the Chinese Clinical Trial Registry (No. ChiCTR-IOR-15007033).

### 2.2. Sample Size

According to the sample size estimation formula for individual randomized controlled trials, a previous literature [19] and the results of our preliminary test (α = 0.05, β = 0.20, *p*_1_ = 11%, *p*_2_ = 3%), a total of 158 participants were needed in each group:

$$N=\frac{\left[Z_{\alpha }\sqrt{2\bar{p}(1-\bar{p})}+Z_{\beta }\sqrt{P_{1}(1-P_{1})+P_{2}(1-P_{2})}\right]}{{\left(p_{1}-p_{2}\right)}^{2}}$$

### 2.3. Population and Procedures

This study population were prediabetics aged 60 years old and over living in rural areas in Yiyang City of Hunan Province. To select a representative sample of prediabetic elderly, a screening program was carried out on the elderly population in Yiyang City. A multistage cluster randomized sampling method was used. In the first stage, sampling was stratified according to geographical characteristics, and two out of six counties were selected. In the second stage, two out of 11 townships, and two out of nine townships were randomly selected. In the third stage, 25% of the rural villages were randomly selected from each chosen township (each township contains 30–50 villages). All households with elderly individuals in each selected villages who had lived in the area for 3 years or longer were eligible to participate prediabetes screening. Those with severe physical and mental illness were excluded from the screening. Individuals who had diabetes were also excluded from the screening. Participants were diagnosed as prediabetic by using oral glucose tolerance tests (OGTT). The diagnostic standards for prediabetes as stated in the 1999 WHO criteria [20] were used and subjects were categorized into three groups: (1) IFG group: fasting plasma glucose of 6.1–7.0 mmol/L (110–126 mg/dL) and a 2-h post-glucose load of <7.8 mmol/L (140 mg/dL); (2) IGT group: fasting plasma glucose of 6.1 mmol/L (110 mg/dL) and a 2-h post-glucose load of 7.8–11.1 mmol/L (140–200 mg/dL); (3) IFG+IGT group. 

More details of the screening procedure and participant characteristics have been described elsewhere [21]. In brief, 2144 elderly took part in the OGTT and 461 elderly individuals had prediabetes. For many reasons, 27 prediabetic elderly were not investigated or refused to take part in the subsequent study, so finally, 434 prediabetics from 42 villages were included in our intervention study.

### 2.4. Baseline Measures

To randomly assign the individuals with prediabetes to the intervention and control groups at baseline, a concealed random allocation method was used. A total of 434 prediabetic subjects were coded according to a random number produced by a computer. The subjects coded as odd numbers were assigned to the control group, while those with even codes were assigned to the intervention group. Finally, 220 prediabetic subjects were assigned to the control group and 214 prediabetic subjects were allocated to the intervention group. 

The socio-demographic information of the 434 prediabetic subjects were collected at baseline by questionnaire and included their age, gender, marriage status, history of hyperglycaemia, family history of diabetes and education. The marriage status was classified as married or non-married. Non-married status included divorce, never-married, losing a partner and living together without a marriage certificate. A family history of diabetes was defined as being present in subjects whose first-degree relatives were diabetic. Education was assessed by asking the participants to select their highest level of education from the following choices: less than 1 year, 1–6 years and 6 years and above.

Food intake and physical activity were quantified at baseline and at each follow-up examination using a standardized interview. The interview covered food consumption over the last 12 months using the CNHS2010-F questionnaire, and the total caloric intake was assessed using the 24-h recall method, with a self-reported food intake frequency and portion size. In addition, the Diet Balance Index (DBI-07) was used to estimate whether they ate a balanced diet (DBI-07 score between 0 and 14). For physical activity, the International Physical Activity Questionnaire (IPAQ-Long version) was used to assess the sitting time and walking time, as well as metabolic equivalents (METs) of the participants during the past week.

The level of plasma glucose was measured using a hexokinase enzymatic method, and the serum lipid levels were assessed enzymatically with commercially available reagents in the biochemical laboratory of the primary care centre in each village. The level of HbA1c was assessed from a fingerstick capillary whole-blood sample using a DCA 2000 analyser. All laboratories had successfully completed a standardization and certification programme. 

Anthropometric measurements were taken while participants were wearing lightweight clothing without shoes. Height was measured to the nearest 0.1 cm by using a stadiometer, and the weight was measured using a Cardinal Detecto digital Scale to the nearest 0.1 kg. The body mass index (BMI) was computed as the weight in kilograms divided by the square of the height in metres (BMI = kg/m^2^). The waist circumference was measured to the nearest 0.1 cm by placing a non-stretching measuring tape horizontally around the participant’s abdomen at the top of the iliac crest. All anthropometric measurements were taken twice and means were used in the analyses. 

### 2.5. Primary Outcome Measures

The development of diabetes was the main outcome, and was determined by assessing the fasting glucose and 2-h plasma glucose level. The fasting glucose and 2-h plasma glucose levels were obtained before and after a 75 g oral glucose load, respectively. If the fasting glucose level was ≥7.8 mmol/L and/or the 2-h glucose level was ≥11.1 mmol/L, then the OGTT test was repeated after 7–14 days. If the results met the standard again, the subject was considered to have reached an endpoint and was suggested to receive diabetes treatment. The OGTT and anthropometric measurements were performed with the same methods at the baseline examination and at intervals of six months.

### 2.6. Intervention 

An Intervention Study Team (IST) was established that included five public health professionals and a registered dietitian to ensure that the intervention was implemented. The team was trained before the intervention was carried out and was responsible for conducting the intervention group sessions and managing participants:

(1) *Standard health advice (control group)*: Subjects were advised to stop smoking, limit the amount of liquor consumed, eat less animal fat and avoid a sedentary lifestyle. These objectives were repeatedly explained every six months by the IST.
(2) *Synthetic intervention (intervention group)*: The synthetic intervention model was formulated through consultations with several experts (12 experts in total) and focus group discussions and included lifestyle education, lifestyle intervention, training for the Self-Monitoring of Blood Glucose (SMBG) and setting up a Help Each Other Group (HEOG) (Table 1).

Lifestyle education: A diabetes knowledge manual and leaflets were designed by our group based on the “Chinese type 2 diabetes prevention guide” and were provided to intervention group participants and their family members every three months via the IST. A 60-min curriculum covering nutrition and physical activity, energy balance, health eating and diabetes knowledge was conducted in every intervention village every three months using a DVD series developed by the IST. 

SMBG: Public health professions trained the prediabetic subjects in the intervention group regarding the correct methods to measure glucose by using a fingerstick glucose detector, which was provided by IST to every participant in the intervention group. They persuaded the participants to monitor their blood glucose of themselves (SMBG), and the subjects reported their measurements to the IST every month. 

Lifestyle intervention: The following instructions were repeated once every three months: (1) Calculate the total calorie requirement: the total calorie requirement of every subject in the intervention group was calculated according to the following formula: Total calories = Ideal body weight (kg) calorie requirement for different physical activity levels per day (kg); where the ideal body weight (kg) = (height (cm) − 100) × 0.9. We defined a “High level of physical activity” as needing 40 kcal per day (per kg), a “Moderate level physical activity” as needing 35–40 kcal per day (per kg), and a “Low level physical activity” as needing 30–35 kcal per day (per kg); (2) Calculate the total caloric intake: The subjects were asked about their usual diet in terms of the amounts and kinds of food they ate using a 24-h recall form for three continuous days. The total caloric intake of the participants was calculated according to the “China Food Composition, Version 2”; (3) If there was no major deviation in the nutrient balance, we emphasized that the everyday diet should remain balanced; (4) If the caloric intake exceeded what they needed, participants with a BMI < 24.0 kg/m^2^ were prescribed a diet containing 25–30 kcal per day per kg and suggested to consume less red meat, animal oils and sugar. Participants with a BMI ≥ 24.0 kg/m^2^ were encouraged to reduce their caloric intake and consume more fruits and vegetables; (5) If the subject was male and his alcohol intake was judged to exceed 25 g or if a female subject’s alcohol intake was judged to exceed 15 g (15 g of alcohol is equal to 450 mL of beer, 150 mL of wine or 50 mL of sake), and consuming alcoholic beverages seemed to be related to them eating more than usual, we advised them to stop consuming alcohol or to reduce the amount by half; (6) Special suggestions were given to participants with malnutrition or who were taking medication(s); (7) For the above instructions on diet, we referred to the “Chinese type 2 diabetes prevention guide, version 2013” [22], which provided a rough gauge of the energy and nutrients; (8) The current physical activity level was assessed by interview, and we used the metabolic equivalents (METs) of subjects for the past week to judge whether the physical activity was sufficient and regular. Subjects who were involved in physical labour or who had to walk or cycle >30 min/day were asked to continue their routine activities. Subjects engaged in sedentary or insufficient physical activity were motivated to walk for at least 30 min every day; (9) A lifestyle (diet and physical activity) counselling session was conducted every three months.

HEOG: The Helping Each Other Group was established according to district. This group contained 5–7 prediabetic subjects who lived close to each other. Responsible people who had no listening or speaking problems and had a better education than the others were appointed to be responsible for motivating the other participants who were engaged in physical activity and diet sessions. 

### 2.7. Motivation and Adherence

Diet and physical activity interventions were implemented every three months. In addition, weekly short message reminders and monthly telephone contacts were maintained by the HEOG responsible persons, as well as other intervention participants, to promote continued motivation. The names and ID numbers were recoded every time when the subjects in the intervention group took part in the lifestyle education curriculum and lifestyle session. An assessment of diet and physical activity adherence was performed every week based on self-reported data using a mobile phone. For diet intervention, the subjects were phoned by the IST to ask about the amounts and kinds of food they ate based on a 24-h recall for the last three days to calculate the calories, and subjects were asked whether they followed the items from (4) to (6). The prediabetic subjects whose total caloric intake did not exceed the caloric intake recommendations and who followed the instructions were considered to be adherent. The other subjects were considered to be non-adherent and were motivated to consume a balanced diet. For the physical activity intervention, subjects who were involved in physical labour or who had to walk or cycle >30 min/day who continued their routine activities were considered to be adherent. Subjects who were engaged in insufficient activities were asked about the times and duration of their walking, and if the number of times was ≥5 and the duration was ≥30 min, they were considered to be adherent.

### 2.8. Statistical Analysis

Continuous variables are performed as mean ± SD and categorical variable are presented as percentages. Comparisons between independent samples used chi-square test for categorical variables, and Student’s *t*-test for quantitative variables. We used least squares multivariate regression to compare the between-group difference for percent change in weight and waist circumference, and absolute change in plasma glucose, weight, BMI, waist circumference, serum lipid and HbA1c. Due to between-group differences in the baseline values of outcome variables could introduce bias from regression to the mean. To minimize the bias, baseline values for the dependent variables were included in each regression models. The differences between the groups in the incidence of diabetes were tested by means of the two-sided log-rank test. Comparing the hazard ratio of subjects developing diabetes between groups, the Cox’s proportional hazard regression model were used. All analysis of endpoints were based on the intention-to-treat principle and performed by using SAS version 9.1 (SAS Institute Inc., Cary, NC, USA). Inferences for comparisons were tested at a 5% two-sided level of significance and missing data were not imputed. 

## 3. Results

### 3.1. Baseline Characteristics

**Figure 1:**
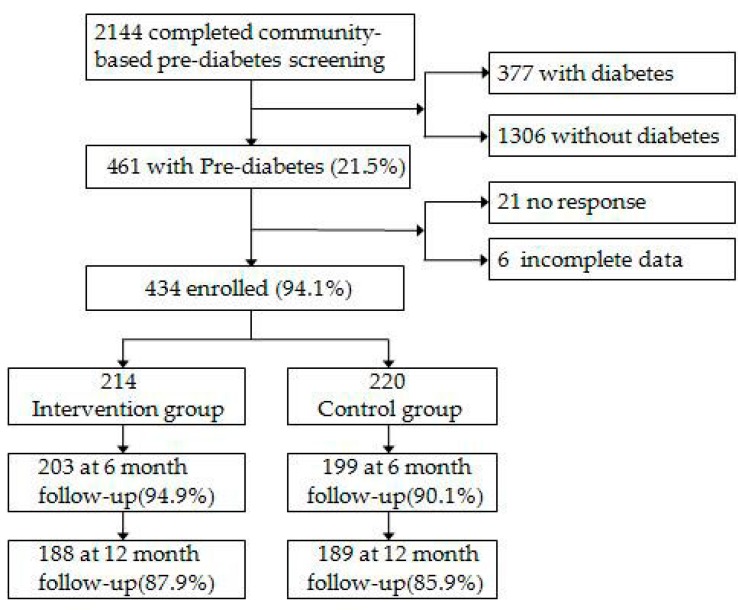
Flow of participants from screening to completion of the final-up assessment.

A total of 2144 participants took part in OGTT for prediabetes screening and 434 prediabetics were included in this follow-up study. Among the 434 participants enrolled in the cohort, 32 (7.4%) dropped out after 6 months, which had no significance between the intervention group and the control group (*p* = 0.08). After 1 year follow-up, a total of 57 (13.1%) had dropped out in the intervention group, and 25 (11.7%) dropped out of the control group 32 (14.5%) dropped. There were no significant differences in the situation of drop out between groups (*p* = 0.377). The enrollment and follow–up experience was depicted in Figure 1. The mean age of 434 pre-diabetes was 69.5 years and there were 180 males and 254 females. The baseline characteristics of the two groups were similar (Table 2). 

### 3.2. Participation and Adherence

Over the 6-month follow-up, 162 (79.9%) subjects attended the education curriculum. The median number of times of SMBG was increased from 0 to 1 (times/month). The proportion of participants who used the self-monitoring was 72.6% in the intervention group. At the 1-year follow-up, 161 (85.7%) subjects attended the education curriculum. During the intervention period, only 127 (67.8%) subjects had completed the education curriculum four times. The median number of times of they performed SMBG was increased from 0 (baseline) to 2.5 (times/month). The proportion of participants who used the self-monitor dropped to 56.7% among the intervention group (Table 3).

At baseline, 35 HEOGs were established, and there were 35 responsible persons. Nobody dropped out during the intervention. During the 6-month follow-up, the proportion of subjects with dietary adherence was 85.6%, and that of physical activity adherence was 54.3%. At the 12-month follow-up, the dietary adherence was 80.2%, and the physical activity adherence was 50.8%. The subjects in the intervention group were more likely to report changes in dietary and exercise habits (Table 3).

### 3.3. Incidence of Diabetes

The incidence of diabetes was 1.5% in intervention group and was 2.5% in control group during 6 months follow-up, but there was no significant difference between groups (*p* = 0.452). After 12 months follow-up, the incidence of diabetes was 4.2% in the intervention group and it was significantly lower than the control group (*p* = 0.041). According to Cox’s proportional hazard regression analysis, the hazard ratio was 0.2 (95% CI: 0.1 to 0.5) after adjusting for age, sex, weight, waist circumference, BMI, physical activity, total calorie consumption, plasma glucose and serum lipid level between the intervention and control group (*p* < 0.001). Results are shown in Table 4.

### 3.4. Outcome at the 1-Year Follow-Up 

Table 5 shows the primary outcome changes after 1-year follow-up. The mean change in fasting plasma glucose was −3.9 mg/dL in the intervention group vs. 2.2 mg/dL in the control group (*p* < 0.001), but there was no clinical meaning in 2 h oral glucose challenge change (*p* = 0.246). Compared to baseline values, percentage of body weight was decreased by 4.5% (95% CI: −6.2% to −2.5%) in the intervention group and increased by 4.6% (95% CI: 2.0% to 7.2%) in the control group (*p* < 0.001). This equated to a mean weight loss of 3.2 kg for intervention participants and a weight gain of 1.7 kg for control participants. 

The waist circumference decreased by 2.9% (95% CI: −3.5% to −2.3%) in the intervention group compared with a 1.3% increase (95% CI: 0.6% to 1.9%) in the control group (*p* < 0.001). This equated to a mean waist circumference decrease of 2.4 cm among the intervention participants versus an increase of 1.0 cm among the control participants. The change in the total cholesterol and HbA1c were also significantly and clinically different between the groups. The total cholesterol decreased by 9.1 mg/dL in the intervention group versus a decrease of 4.6 mg/dL in the control group (*p* = 0.014). The HbA1c decreased 1.0 mg % among the intervention participants versus an increase of 0.1 mg % among the subjects in the control group (*p* = 0.002).

## 4. Discussion

This study provides evidence that type 2 diabetes among elderly individuals with prediabetes in rural China can be prevented by synthetic intervention. The crude diabetes incidence in the intervention group was 4.2%, but was 19.7% in the control group at the end of one year (*p* = 0.041). The risk of incident diabetes among the intervention participants was 0.2 times lower than that of control participants (*p* < 0.001). The results of our study were similar to those of previous studies and showed that lifestyle intervention could reduce the incidence of diabetics [23,24]. It should be noted that the progression rate of prediabetes to diabetes was very high in the elderly population, as shown by an incidence of 19.7% after one year in the control group. This was higher than the rate in previous studies [18,25]. The high rate of progression to diabetes in our population might be related to age, which was also observed in several previous studies [26,27]. However, our subjects also had some differences compared with other studies, such as the DAQING and DPP studies, in that some subjects had persistent IGT at the time of recruitment. In our study, 13.4% of the subjects had IGT+IFG, and the conversion rate of IGT+IFG to diabetes was previously shown to be three times higher than IGT alone [26]. Moreover, the low income in rural areas may be another reason. Based on our face-to-face interviews conducted during this study, many elderly people expressed that they were afraid to get sick because they had no money, so they may be more likely to try to do something to keep themselves from getting sick.

Compared to other intervention studies, there were some obvious differences that should be considered when evaluating the results. First, all subjects were trained on the correct method to perform self-monitoring of blood glucose (SMBG) by professionals. There have been several studies that have evaluated the usefulness of SMBG on glycaemic control, which showed that SMBG was associated with decreased diabetes-related morbidity and all-cause mortality in T2D cases [28,29]. However, it has been reported that there is a low utilization of SMBG in Chinese adults with T2D [30], and the persistence of SMBG in this study was unknown due to the low health literacy and heavy economic burden in the subjects [21]. Second, apart from diet and physical activity intervention, the HEOG played an important role in preventing diabetes among prediabetic elderly subjects. Some previous studies found that community health workers (CHWs) who played a role as liaisons between healthcare providers and community members improved the subjects’ health [31,32]. However, in this study, the HEOG were more effective than the CHWs. This is not surprising because in a previous study, the HEOG were shown to have more daily communication and discussions about trivial affairs and were part of healthy living partnerships [33], which allowed them to improve the motivation of workers, especially in rural areas lacking health resources.

The most important observation of this intervention study was the reduction in plasma glucose among elderly individuals with prediabetes in the intervention group over the 12-month period. Our study revealed that the fasting glucose decreased by 3.9 mg/dL in the intervention group after one year. This effect compared favourably with other studies. For example, the DPP study [8] reported that the fasting glucose decreased by 5 mg/dL after 2.8 years of follow-up, and the DPS study [9] conducted in Finland that involved 522 middle-aged, overweight people with elevated glucose demonstrated that the fasting glucose decreased by 4 mg/dL after 3.2 years.

The effect of this intervention on adiposity was also comparable with other studies. The most recent meta-analysis including 25 studies showed that exposure to lifestyle interventions resulted in a mean 2.32 kg weight loss after 12 months [34]. The DEPLOY Pilot Study [35] reported that the weight loss was −5.7 kg (−6%) and the BMI had decreased 5.8% at 12 months. Similarly, the HELP PD study [36] reported that the weight decreased 7.1 kg (−7.3%), the BMI decreased 2.1 kg/m^2^ and the waist circumference was reduced by 5.9 cm after 12 months. The participants in our intervention group lost an average of −3.2 kg (4.5%), their waist circumference was reduced by 2.4 cm (2.9%) and the BMI decreased by 3.2 kg/m^2^ at the end of one year. The differences in the results may be due to the different target populations. The subjects in the DEPLOY and HELP PD studies had a BMI ≥ 24 kg/m^2^ and age <60 years, but our study population had an average BMI < 24 kg/m^2^ and age ≥60 years.

It may seem remarkable that the subjects in the control group gained body weight and had increases in their BMI and waist circumference. However, a previous study of the elderly in rural China showed that they had a poor education and health literacy [21], and unhealthy lifestyle behaviours were prevalent [37,38]. Even though we gave standard health advice to the subjects in the control group, the suggestions were not followed or were not accepted. In addition, the aging of the entire fixed cohort may have contributed to the natural weight gain, especially in an elderly population with prevalent total obesity at baseline (12.7%).

The synthetic intervention model used in our study not only improved the glucose tolerance of the subjects but also reduced the magnitude of several cardiovascular risk factors. The TC decreased 9.1 mg/dL in the intervention group versus 4.6 mg/dL in the control group (*p* = 0.014). The HbA1c decreased 1.0 mg % among intervention participants and increased 0.1 mg % in the control group (*p* = 0.002). Nevertheless, because of the differences between research methods and target populations, the final results were somewhat divergent. For example, Kramer et al. [39] found that the HDL-C and TC levels were not clinically different between the intervention and control groups among 93 non-diabetic individuals with a BMI ≥ 25.0 kg/m^2^. Whittemore et al. [40] demonstrated a marginal trend for higher HDL levels in the intervention group at six months compared to controls. Wolf et al. [41] found that the HbA1c level was deceased 0.59 mg % at four months after starting the intervention, but there was no significant difference at 12 months. 

The effect of the intervention was assessed after one year in our study because earlier assessments may have been biased because the subjects were conscious of being studied. Moreover, our estimated effects of the intervention can be considered conservative for two reasons. First, the data were analysed according to an intention-to-treat principal, even though some participants in the intervention group did not follow the diet and exercise programmes that we recommended. Second, all participants received general advice on improving their health at the baseline and follow-up visits, and they were encouraged to pay attention to their health. Even so, self-reported information on diet and physical activity adherence may overestimate the effect size on the results because some participants in the intervention group may have reported higher adherence than they actually had.

Our study has several limitations. First, the attrition of this cohort was not addressed, although the rate was low (12.9%), while some studies stated that missing data under a missing-at-random (MAR) assumption were more plausible [42,43]. To decrease the impact of cohort attrition on the available data, further follow-up studies should be conducted. Second, the study lacked generalizability and long-term maintenance. All participants were elderly individuals with prediabetes and lived in rural areas, therefore, our study results may not be applicable to younger individuals with prediabetes and those living in urban areas. Third, we did not separately analyze the effectiveness of the synthetic intervention model for the three different categories of prediabetes, but a previous meta-analysis showed that the annualized incidence of diabetes for isolated IGT was 5.52% and that for isolated IFG was 4.66%, which were lower than those for IFG and IGT combined (12.13%) [44]. Finally, our investigators collected the outcome at the end of one year. However, our study was not a blinded study, which may create potential interviewer bias due to investigators’ longing for the lifestyle intervention to be proven effective.

## 5. Conclusions

Our study evaluated the effectiveness of one year’s synthetic intervention on preventing diabetes among elderly individuals with prediabetes in rural China. The synthetic intervention contributed to weight loss and fasting glucose decrease, which may be effective in preventing diabetes among elderly individuals with prediabetes in rural China. Further follow-up studies should be conducted to evaluate the long term benefits of T2D prevention after the synthetic intervention implementation.

## Figures and Tables

**Table 1:** Synthetic intervention model.

Time Interval	Intervention Components	Intervention Content
3 months	Lifestyle education	1. Giving out diabetes knowledge manual and leaflets
		2. Education curriculum
3 months	Lifestyle intervention	1. Giving instructions about diet and physical activity
		2. lifestyle counsel sessions
3 months	SMBG	1. Training method of fasting glucose measurement
		2. Reporting SMBG times
At first intervention	HEOG	1. Setting HEOG
		2. Choose HEOG responsible persons

**Table 2:** Baseline characteristics of the subjects in the intervention and control group.

Variables	Intervention Group	Control Group	Value *x*^2^ or *t*	*p* Value
n	214	220		
Age (years)	69.2 ± 6.8	69.5 ± 6.3	0.38	0.67
Sex				
Male	93 (43.5)	87 (39.5)	0.68	0.41
Female	121 (56.5)	133 (60.5)		
Marriage status				
Married	146 (68.2)	166 (75.5)	2.81	0.09
Non-married	68 (31.8)	54 (24.5)		
Education				
Less than 1 year	35 (16.4)	46 (20.9)	2.47	0.29
1–6 years	134 (62.6)	138 (62.7)		
6 years and above	45 (21.0)	36 (16.4)		
History of hyperglycemia *				
Yes	11 (5.1)	17 (7.7)	1.20	0.27
No	203 (94.9)	203 (92.3)		
Family history of diabetes				
Yes	14 (6.5)	22 (10.0)	1.71	0.19
No	200 (93.5)	198 (90.0)		
Weight (kg)	59.5 ± 8.8	59.7 ± 10.6	0.63	0.53
BMI (kg/m^2^)	23.5 ± 3.2	23.9 ± 3.7	1.73	0.09
Waist circumference (cm)	84.2 ± 9.8	85.6 ± 8.8	1.73	0.09
Plasma glucose (mg/dL)				
Fasting	111.0 ± 10.9	109.8 ± 8.8	1.38	0.17
2-h glucose	153.9 ± 18.9	156.5 ± 20.4	1.41	0.16
Serum lipid (mg/dL)				
Total cholesterol	99.8 ± 12.8	99.0 ± 12.4	0.65	0.51
HDL-C	32.3 ± 9.4	31.7 ± 7.9	0.80	0.43
Triglycerides	36.8 ± 20.3	34.8 ± 21.1	1.02	0.31
HbA1c (mg %)	5.7 ± 0.9	5.8 ±1 .1	0.95	0.34
Physical activity (MET/week)	1484.0 ± 959.4	1382.8 ± 925.4	1.12	0.26
DBI-07 score				
≤14	46 (21.5)	64 (29.1)	3.31	0.07
≥15	168 (78.5)	156 (70.9)		
Total kcal/day	1763.6 ± 235.6	1784.2 ± 213.2	0.95	0.34

Data were described as *n* (%) or means ± SD, *t* test were used for continuous variables and *x*^2^ test was used for categorical variables; * History of hyperglycemia: A situation of fasting glucose >6.1 mmol/L or 2-h glucose >7.8 mmol/L without diagnosis of diabetes was measured in other places before this interview.

**Table 3:** Participation, adherence and health-related behaviors changes during the intervention.

Variable	Follow Up 6 Months Intervention Control		Follow Up 12 Months Intervention Control	
**Lifestyle Education**				
Attended an Education Curriculum	162 (79.9)	-	161 (85.7)	-
**Lifestyle Intervention**				
Decreased Consumption of Fat	93 (45.8)	49 (24.6) ^†^	156 (83.0)	56 (29.6) ^†^
Increased Consumption of Vegetables	78 (38.4)	30 (15.1) ^†^	98 (52.1)	32 (16.9) ^†^
Increased Consumption of Fruit	30 (14.8)	25 (12.6)	50 (26.6)	43 (22.8)
Decreased Consumption of Alcohol	43 (21.2)	40 (20.1)	55 (29.3)	44 (23.3)
Decreased Consumption of Sugar	168 (82.8)	110 (55.3) ^†^	142 (75.5)	105 (55.6) ^†^
Decreased Calorie Intake	86 (42.4)	47 (23.6) ^†^	75 (39.9)	28 (14.8) ^†^
Increased Walking Time	76 (37.4)	40 (20.1) ^†^	98 (52.1)	42 (22.2) ^†^
Decreased Sitting Time	49 (24.1)	33 (16.6)	57 (30.3)	35 (18.5) ^†^
**SMBG**	147 (72.6)	-	107 (56.7)	-

Data were presented as *n* (%); ^†^: *p* values were determined by the chi-square test for the difference between the groups.

**Table 4:** Cumulative incidence of diabetes at 6 and 12 months follow-up.

Variable	Intervention Group	Control Group	*p*-Value
**At 6 months**			
*n*	203	199	
Incidence of diabetes ^†^, % (95% CI)	1.5 (0.0, 3.4)	2.5 (0.5, 5.0)	0.452
Adjusted HR ^††^ (95% CI)	0.6 (0.1, 2.6)	1.0	0.509
**At 12 months**			
*n*	188	189	
Incidence of diabetes,% (95% CI)	4.2 (1.5, 6.7)	19.7 (13.8, 25.1)	0.041
Adjusted HR ^††^ (95% CI)	0.2 (0.1, 0.5)	1.0	<0.001

^†^: The incidence of diabetes was tested by means of the two-sided log-rank test; ^††^: Diabetes and survival time as dependent variables and intervention as independent variable for Cox regression, adjusted for age, sex, weight, waist circumference, BMI, physical activity, total calorie, plasma glucose and serum lipid level.

**Table 5:** Changes in selected primary outcomes and metabolic variables from baseline to the end of year 1 in the subjects in the intervention and control group.

Outcome	Intervention Group	Control Group	*p*-Value
Change in Weight			
In kilograms	−3.2 (−4.2, −2.3)	1.7 (0.2, 3.1)	<0.001
Percent change	−4.5 (−6.2, −2.5)	4.6 (2.0, 7.2)	<0.001
Change in BMI (kg/m^2^)	−3.2 (−3.8, −2.9)	0.8 (0.2, 1.4)	<0.001
Change in Waist circumference			
In centimeter	−2.4 (−3.0, −1.9)	1.0 (0.4, 1.5)	<0.001
Percent change	−2.9 (−3.5, −2.3)	1.3 (0.6, 1.9)	<0.001
Change in glucose (mg/dL)			
Fasting	−3.9 (−6.2, −1.8)	2.2 (0.6, 3.8)	<0.001
2-h glucose	−8.7 (−12.8, −4.7)	−5.4 (−9.2, −1.2)	0.246
Change in serum lipid (mg/dL)			
Total cholesterol	−9.1 (−11.7, −6.1)	−4.6 (−7.3, −2.1)	0.014
HDL-C	3.4 (1.6, 5.3)	2.4 (1.0, 4.0)	0.415
Triglycerides	−4.2 (−7.9, −0.5)	−0.7 (−4.6, 3.2)	0.199
Change in HbA1c (mg %)	−1.0 (−1.2, −0.8)	0.1 (−0.1, 0.4)	0.002

Data were presented at mean (95% confidence interval), *p*-values represent the between-group comparison of the average of baseline with 12 mouths adjusted baseline values in regression model.
